# Genetic Analysis of the Fatty Acid Profile in Gilthead Seabream (*Sparus aurata* L.)

**DOI:** 10.3390/ani11102889

**Published:** 2021-10-03

**Authors:** Antonio Vallecillos, María Marín, Martina Bortoletti, Javier López, Juan M. Afonso, Guillermo Ramis, Marta Arizcun, Emilio María-Dolores, Eva Armero

**Affiliations:** 1 Department of Agronomic Engineering, Technical University of Cartagena, Paseo Alfonso XIII 48, 30202 Cartagena, Spain; antonio.vallecillos@edu.upct.es (A.V.); mirenamm@gmail.com (M.M.); jlaela2@hotmail.com (J.L.); emilio.mariadolores@carm.es (E.M.-D.); 2 Department of Comparative Biomedicine and Food Science (BCA), University of Padova, Viale dell’Universitá 16, 35020 Legnaro, Italy; martina.bortoletti@studenti.unipd.it; 3 Institute of Sustainable Aquaculture and Marine Ecosystems (GIA-ECOAQUA), Carretera de Taliarte s/n, 35214 Telde, Spain; juanmanuel.afonso@ulpgc.es; 4 Department of Animal Production, University of Murcia, Avenida Teniente Flomesta 5, 30860 Murcia, Spain; guiramis@um.es; 5 Spanish Institute of Oceanography, Oceanographic Center of Murcia, Carretera de la Azohía s/n, 30860 Puerto de Mazarrón, Spain; marta.arizcun@ieo.es

**Keywords:** fatty acid profile, heritability, gilthead seabream, body weight, moisture, fat, collagen, protein

## Abstract

**Simple Summary:**

Humans require essential fatty acids in their diet and marine fish are a source of them, especially omega3 fatty acids that present high benefits on diverse vascular diseases and the immune system. Breeding programs in gilthead seabream usually include growth as the first criterion in the selection process of the fish. However, that could lead to fish with a higher fillet fat content and a fatty acid profile with a lower polyunsaturated fatty acids percentage. Fillet fat content and its fatty acids profile have been revealed as heritable traits. Therefore, further studies to go deeper in the selection process are advisable.

**Abstract:**

The gilthead seabream is one of the most valuable species in the Mediterranean basin both for fisheries and aquaculture. Marine fish, such as gilthead seabream, are a source of n3 polyunsaturated fatty acids, highly appreciated for human food owing to their benefits on the cardiovascular and immune systems. The aim of the present study was to estimate heritability for fatty acid (FA) profile in fillet gilthead seabream to be considered as a strategy of a selective breeding program. Total of 399 fish, from a broodstock Mediterranean Sea, were analysed for growth, flesh composition and FA profile. Heritabilities for growth traits, and flesh composition (fat, protein, and moisture content) were medium. Heritability was moderate for 14:0, 16:0 and 18:1n9 and for sum of monounsaturated FA and n6/n3 ratio, and it was low for 20:1n11 and 22:6n3 and the ratio unsaturated/saturated FA. Breeding programs in gilthead seabream usually include growth as the first criterion in the selection process of the fish. However, other quality traits, such as fillet fat content and its fatty acids profile should be considered, since they are very important traits for the consumer, from a nutritional point of view and the benefits for the health.

## 1. Introduction

The gilthead seabream is one of the most valuable species in the Mediterranean basin both for fisheries and aquaculture. Total production in Mediterranean countries reached 253,000 metric tons in 2019. The three most important countries producing gilthead sea bream were Greece, Turkey and Spain, in that order [1]. In the Mediterranean area, notable success has been achieved in the production of diverse species, such as sea bream and bass. Now that production technologies have been established, interest has been redirected to increasing the quality of the product offered [2].

Fatty acids (FA), especially n3 polyunsaturated fatty acids (n3 PUFA), eicosapentaenoic (EPA, 20:5n3) and docosahexaenoic acids (DHA, 22:6n3), are recognised as being beneficial for human health, controlling a wide range of human pathologies including cardiovascular, diabetes, rheumatoid arthritis, osteoporosis, asthma, cognitive decline, neurological dysfunction and possible cancers, whilst also playing an important role in neural development and in the immune and inflammatory processes [3,4,5,6]. Arachidonic acid (20:4n6, ARA), EPA and DHA are considered as the precursors for the synthesis of eicosanoids, regulators of cell signalling and gene expression and the most powerful modulators of cell membrane fluidity [7,8]. Alteration in the PUFA content of the immune cells was demonstrated to be associated with the alteration of non-specific immunity (e.g., phagocytosis, respiratory burst and serum lysozyme), specific immunity (e.g., antibody production and resistance to pathogens), eicosanoid production and immune-related gene expression [9].

Humans, and probably all vertebrates, require essential FA in their diet that cannot be biosynthesised or interconverted, such as 18:2n6 (linoleic acid, LA) and α18:3n3 (αlinolenic acid, ALA) PUFA [10,11,12]. These essential fatty acids are primarily derived from plants that should be included in the vertebrates’ diet. LA and ALA have vital functions in themselves, and in turn act as precursors for the long-chain PUFA (LCPUFA) ARA, EPA and DHA. Their biosynthesis from LA and ALA can be carried out by mammals, although the process of EPA and particularly DHA biosynthesis from ALA is very low in humans and in marine fish [6,13]. The biosynthetic pathway involves consecutive desaturation and elongation reactions that convert LA to ARA and ALA to EPA and DHA. The two main enzyme families involved in these conversions are the elongases of very long fatty acids (Elovl) and the fatty acyl desaturases (Fad) [6]. The EPA pathway in teleost fish is often incomplete, primarily due to a lack of Δ5 desaturase, and so synthesis of EPA from ALA is not possible in many, particularly marine, carnivorous species [14]. However, the DHA pathway from EPA is probably functional in most teleost fish, including marine species, at least in some tissues.

Modern Western diets have an excess of n6 PUFA, primarily LA, and because n6 FA and n3 FA cannot be interconverted in vertebrates, this has led to an increase in the tissue ratio of n6 to n3 LCPUFA, linked to cardiovascular, inflammatory, and neurological problems [7]. One way of addressing this n6/n3 imbalance is to increase the levels of n3 PUFA and especially n3 LCPUFA in the diet of humans. Marine fish, such as the gilthead seabream (*Sparus aurata*), are a source of LCPUFA [15]. However, vegetable feed in the fish diet has increased in recent years. Fillets of gilthead sea bream fed diets rich in plant oils show increased levels of LA and ALA, with a concurrent decrease in EPA and DHA [16,17].

Dietary and fillet FA composition are closely associated. However, Ballester-Lozano et al. [18] observed that the FA composition (%) depends not only on the diet but also on the fillet lipid content (FLC). In general, monounsaturated FA (MUFA) increased with the increase of FLC, whereas the trend for saturated FA (SFA) and PUFA was the opposite. In the case of ARA and DHA, they decreased when the FLC increased. Thus, FLC could partly explain saturated, monounsaturated and some polyunsaturated (ARA and DHA) FA but not LA, ALA, EPA and docosapentaenoic acid (22:5n3, DPA). This is likely due to marine fish showing a limited ability to convert C18 FA into LCPUFA of n6 and n3 series [19,20]. In addition, while triacylglycerols (TAG) are in fat deposits, and usually contain MUFA (C16-C18); polar lipids, in membrane cells are composed mainly of phospholipids that accumulate LCPUFA [21]. An FLC increase is related to a higher amount of fat deposits and, concurrently, a higher proportion of TAG and MUFA and a lower proportion of polar lipids and LCPUFA.

Other factors besides diet (e.g., genotype, gender, age, and production system) have a significant influence on the fillet lipid level and thus on the FA composition of most animals [22].

Hence, advances in breeding programs are essential to contribute to the profits and competitiveness of the companies, as well as to improve the quality of product, including its fatty acid profile.

Selective breeding programs have been initiated in gilthead seabream to improve growth performance and morphology traits; other objectives (feed efficiency, product quality and disease resistance) have been considered later [18,23,24,25]. Heritabilities, genetic and phenotypic correlations between different traits are key indicators in the success of such breeding programs.

To date, there have been few heritability studies in terms of the fillet fat content in gilthead seabream, in which medium heritability in fish and medium correlation with respect to weight were observed [26,27]. In addition, to the best of our knowledge, there has been no work about FA genetic variation in sea bream, but it has been studied in Atlantic salmon [28] and in Nile Tilapia [29]. Flesh n3 LCPUFA composition was a highly heritable trait in Atlantic salmon [28] however individual FA heritability varied from zero to medium in Nile tilapia [29]. In Atlantic salmon, families with a high percentage of n3 LCPUFA in flesh showed higher expression of lipid transport genes, cell cycle, and growth-related genes and increased activity of a transcription factor, hepatic nuclear factor 4α (HNF4 α). Dong et al. [30,31] demonstrated that HNF4α is a transcription factor of the vertebrate *Fad* gene involved in the transcription regulation of LCPUFA biosynthesis. Therefore, it is nonetheless a sensible strategy to select for this trait to improve it and optimise the efficiency of n3 LCPUFA metabolism and flesh levels, irrespective of likely dietary levels.

The aim of the present study was to estimate genetic parameters for the FA profile in fillet sea bream, for the first time in gilthead seabream, to be considered as a strategy in a selective breeding program.

## 2. Materials and Methods

To ensure that animal welfare standards are maintained, anaesthetic was used within the sampling procedure. All animal experiments described in this manuscript fully comply with the recommendations in the Guide for Care and Use of Laboratory Animals of the European Union Council (2010/63/EU) and, whenever necessary, fish were anesthetized.

### 2.1. Fish and Rearing Conditions

For growth, flesh composition (fillet fat, moisture, protein, and collagen percentages) and FA profile were analysed for 399 gilthead seabream fish. The fish came from a broodstock (*n* = 133; 57 males and 76 females) that had been captured in the Mediterranean Sea and maintained in Instituto Español de Oceanografía, Mazarrón, Murcia (IEO). The broodstock had never been subjected to genetic selection.

In the broodstock, the female/male ratio was approximately 2:1 in the tanks, they were under a controlled photoperiod (8L:16D) to synchronize maturation; and egg release was initiated at the beginning of February 2016. During that period, the animals were fed on Vitalis Cal (Skretting, Stavanger, Norway), and egg production was monitored daily. When the total egg production stabilized, one egg batch was established at the end of April 2016. Therefore, eggs from the broodstock were collected and pooled for four consecutive days (4 DL model) to maximize family representation. Incubation was carried out in cylindrical conical tanks (1000 L) at a density of 500–1000 larvae/L. Water conditions were as follows: Temperature 19.0 °C, salinity 34‰, and dissolved oxygen was 6.4 mg/L.

At 251 days post-hatching (dph), the fish were individually tagged in the abdominal cavity for individual identification with passive integrated transporter (PIT; Trovan Daimler-Benz, United Kingdom), following the tagging protocol described by Navarro et al. [32]; initial body weight (BW_251dph_) and total length (TL_251dph_) were measured; and a sample of caudal fin was collected and preserved in absolute ethanol at room temperature for future DNA extraction. Ten days later, the fish were moved to the facilities of the company Servicios Atuneros del Mediterraneo S.L. (San Pedro del Pinatar, Murcia, Spain), where they were reared in a cage in the Mediterranean Sea under intensive conditions: a cage 11 m in diameter which is anchored at a depth of 38 m in the Mediterranean Sea (average water temperature = 18.2 ± 0.9 °C, dissolved oxygen: 7.4 mg/L, 100% oxygen-saturation, salinity: 37.9‰; data estimated from open sea conditions). Fish were fed over the course of the study with extruded pellets (Dibaq S.A, Fuentepelayo-Segovia, Spain), with two different commercial diets. The first 15 months diet D4 was used (46.5% protein, 19% fat, 7% ashes, 2.75% cellulose, 17.9 MJ/kg digestible energy), and subsequently when the fish were around 220 g in weight, they were fed diet D6 (44% protein, 20% fat, 7.17% ashes, 3.07% cellulose, 17.6 MJ/kg digestible energy) until slaughter time. The FA composition of each diet was analysed in duplicate; the mean is shown in Table 1.

At harvest size (980 dph), fish were slaughtered by immersion in ice cold water (hypothermia); final body weight (BW_980dph_) and total length (TL_980dph_) were measured. Fish were manually skinned and filleted without including the nape and the belly flap. Two pieces of fillet were vacuum packaged and frozen at −80 °C for further analysis.

### 2.2. Flesh Composition Quality

One piece of fillet from each fish was homogenized and analysed by indirect method of near-infrared spectroscopy (near infrared spectroscopy, NIR), using FOODSCAN LAB equipment (FOSS IBERIA, Barcelona, España), to obtain total collagen of the muscle, and chemical components of the muscle (fat, moisture, and protein), as a percentage of flesh.

### 2.3. Chromatographic Analysis of Fatty Acid Methyl Esters

Fatty acid methyl esters (FAMEs) were prepared using a solution of KOH in methanol [33], 17:0 acid was used as internal standard FA, then separated and analysed by gas chromatography. Analyses were performed on a 6890-gas chromatograph (Agilent Technologies, Palo Alto, CA, USA) equipped with a GERSTEL MultiPurpose Sampler (MPS2) and a mass spectrometer 5975 with a hyperbolic quadrupole (Agilent Technologies, Palo Alto, CA, USA). Extract (0.8 μL) of FAME was injected and separated on a DB-23 capillary column (Agilent Technologies, Palo Alto, CA, USA) of 60 m (length) × 0.25 mm (internal diameter) × 0.25 µm (film) in constant pressure mode. Chromatographic-grade helium was used as the carrier gas. The temperature of the injector was 240 °C. The inlet operated in split mode with a split ratio of 1:20. The initial oven temperature was 50 °C which was held for 1 min, then increased to 175 °C at 25 °C per min and thereafter increased to 235 °C at 4 °C per min, with a holding time of 10 min. Mass spectra were collected in the scan range *m*/*z* 40–400. The measurements were performed using an electron bombardment ion source with electron energy of 70 eV. The transfer line, source, and quadrupole temperatures were set at 280, 230, and 150 °C, respectively. The chromatograms and mass spectra were evaluated using the ChemStation software (G1791CA, Version D.03.00, Agilent Technol.). Peaks were identified by comparison of retention times with FAME standards (Supelco 37 Component FAME mix, Sigma Aldrich, St. Louis, Missouri, USA) and their mass spectra. The individual FAs are expressed as a percentage of the total FA detected.

### 2.4. Microsatellite Genotyping and Parental Assignment

The broodstock and offspring were genetically characterised. To this end, DNA was extracted from the caudal fin using the DNeasy kit (QIAGEN^®^, Hilden, Germany), and then kept at 4 °C. Next, DNA quantity and quality were determined with a NanoDrop™ 2000 spectrophotometer v.3.7 (Thermo Fisher Scientific, Wilmington, NC, USA). The multiplex SMsa1 (Super Multiplex *Sparus aurata*) was used as described in [34] for genotyping the broodstock and offspring. The electropherogram was analysed using Microsatellite analysis cloud (Thermo Fisher Scientific, Waltham, MA, USA). Direct count of heterozygosity in the offspring was calculated with the Excel package called Gene Alex [35]. For parental assignment, the exclusion method as implemented in VITASSING (v.8_2.1) software [36] was used. The number of fish assigned to a single couple was 399 and they were used to estimate the genetic parameters.

### 2.5. Statistical Analysis

All data were tested for normality and homogeneity of variances using SPSS (v.25.0) [37]. For growth trait (BW and TL) arithmetic means and standard errors were calculated.

Flesh composition (fillet fat, moisture, protein, and collagen percentages) and FA profile were analysed with the following general linear model (GLM):

Y_ij_ = µ + b*covariate_j_ + e_ij_ (1)

in which,

Y_ij_ is an observation of an individual j from the origin i,

μ is the overall mean,

b is the regression coefficient between the analysed variable and the covariate *BW* for flesh composition or fillet fat percentage for FA profile,

e_ij_ is a random residual error.

The level of significant difference was set at *p* < 0.05.

Genetic parameters were estimated under a Bayesian approach using a bivariate mixed model. The model was,

*Y* = *Xβ* + *Zu* + *e* (2)

where *Y* is the recorded data on the studied traits, *β* includes covariate body weight (not included for BW and TL traits), *u* the random animal effect and *e* the error. This was performed using gibbs3f90 program for all traits, as developed by Misztal et al. [38]. The analysis was carried out between two traits each time. The following multivariate normal distributions were assumed a priori for random effects:

*p*(β) ~ k; *p*(u|G) ~ (0; G⊗A); *p*(e|R) ~ (0; R⊗A); (3)

where A is the relationship matrix and k is a constant,

(4) $$G=\left[\begin{array}{cc} \sigma_{u1} & \sigma_{u1,u2}\\ \sigma_{u2,u1} & \sigma_{u2} \end{array}\right],\;R=\left[\begin{array}{cc} \sigma_{e1} & \sigma_{e1,e2}\\ \sigma_{e2,e1} & \sigma_{e2} \end{array}\right].$$

Bounded uniform priors were assumed for the systematic effects and the (co)variance components (G, A). A single chain of 200,000 iterations was run. The first 50,000 iterations of each chain were discarded, and samples of the parameters of interest were saved every five iterations. Density plots to represent posterior marginal distribution of heritabilities, posterior means (PM) and the 95% interval of the highest posterior density (HPD 95%) were obtained through R Development Core Team [39].

## 3. Results and Discussion

### 3.1. Phenotyping

The phenotypic results for growth at 251 dph and 980 dph (BW and TL), flesh composition (fat, collagen, moisture and protein percentages) and FA profile at 980 dph in gilthead seabream are shown in Table 2 and Table 3.

Regarding flesh composition, the fat percentage was high in comparison with that found by other authors [26,27] who observed less fillet fat percentage when BW was lower (In García-Celdrán et al. [26] 4.64% for BW_690dph_ = 271 g, in Elalfy et al. [27] 6.55% for BW_700dph_ = 313 g). However, when fish were raised in an estuary [27] and reached higher BW_700dph_ (440 g), the fillet fat percentage increased (8.71%). In addition, a pronounced seasonality has been observed on fillet fat that reached a maximum with the replenishment of body fat stores in early autumn [18] when our fish were slaughtered. In addition, BW had a positive significant effect on fat percentage, and this effect was less pronounced for collagen percentage (when fish weight increased 100 g the fat percentage increased 0.6% and collagen percentage decreased 0.1%). Contrary to the fat, in our study the moisture percentage was low in comparison with that in García-Celdrán et al. [26] (73%) and Elalfy et al. [27] (73.1% in the cage and 68.8% in the estuary) and BW had a negative significant effect on moisture (when fish weight increased 100 g the moisture percentage decreased 0.7%). This result was logical due to the high negative correlation between fat and moisture [25,26].

Gilthead seabream fillet showed the highest percentage of MUFA, followed by PUFA and SFA with similar percentages (Table 3). Fillet FA composition was closely related to the diet composition but not totally. In fact, in comparison with the diet, fillet showed higher SFA especially for 16:0, and lower MUFA and PUFA percentages, mainly due to the lower percentages of 18:1n9, 18:2n6 and 18:3, although for 22:6n3 the percentage increased notably in comparison to the diet. This difference in FA composition is largely explained by variations in the level of fattening, especially intramuscular fat, the percentage of PUFA, one of them DHA, decreased when FLC increased [40]. In our study, although the fish showed high FLC, the BW was much lighter than in Ballester-Lozano et al. [18] and it is likely that they had not finished their development and fat deposition, and concurrently the DHA was high.

The main fillet FA were 18:1n9, 16:0, LA, and DHA, in accordance with that described by other authors [18,41,42].

### 3.2. Microsatellite Genotyping and Parental Assignment

The use of multiplex SMSa1 PCR using the exclusion method, with a maximum of two tolerated errors, provided successful parental assignment for 91.4% of the offspring. After the assignment, six out of 76 females contributed with 52.1% of the offspring and 29 females did not produce any offspring, whilst six out of 57 males contributed with 60.9% of the offspring and 19 males did not contribute. Pedigree construction using selected highly informative microsatellite markers yielded 66 full-sib families with a mean of 3.86 sibs (range 2–28 sibs).

Regarding the study of genetic variation considering the microsatellites genotypes, high heterozygosity was observed, reaching 0.75. This value is consistent with the fact that the population came from a broodstock that had never been subjected to selection, and reveals that, at that moment, there was no danger of inbreeding.

### 3.3. Genetic Parameters

#### 3.3.1. Heritability for Growth Traits

**Figure 1:**
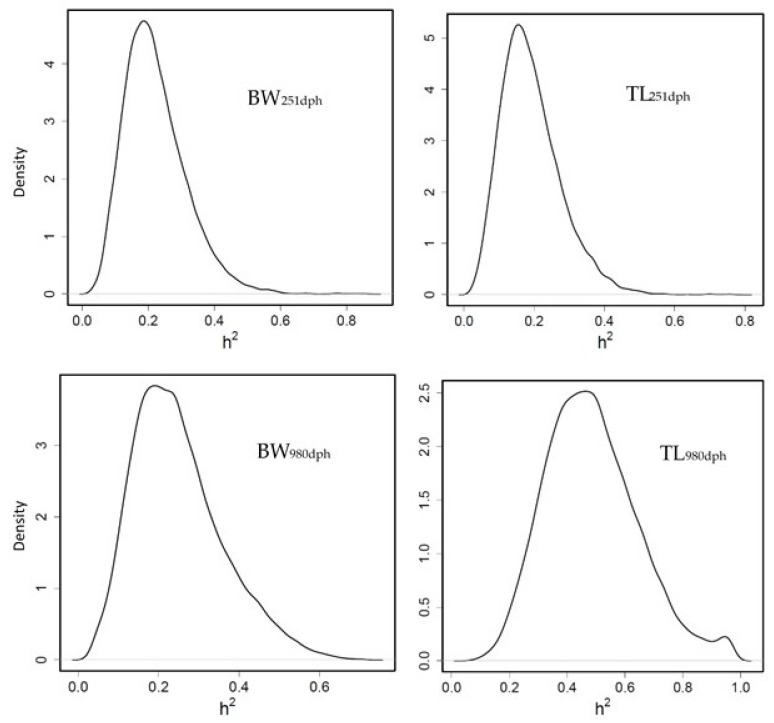
Posterior marginal distribution of heritabilities of body weight (BW) and total length (TL) gilthead seabream. h2 = heritability (*n* = 399).

Heritability for BW_251dph_ and BW_980dph_ was moderate (PM = 0.22 and HPD = [0.06–0.40]; PM = 0.24 and HPD = [0.06–0.48] respectively). For TL, heritability was moderate (0.19 [0.04–0.36] at 251 dph and high (0.48 [0.18–0.80]) at 980 dph (Figure 1), in accordance with other authors [24,26,42].

In our study, TL at advanced age was presented as more heritable than BW; however, other studies [24,26,42] observed similar heritability for both traits and high genetic correlation between them, as also happened in our study (0.94 ± 0.06 genetic correlation BW-TL_980dph_). In addition, García-Celdrán et al. [43] pointed out that heritability estimates for growth traits increased with age when they compared juveniles with commercial size fish. In our study, genetic correlation (rg) for BW or TL at different age were practically null but a safe interpretation of the rg is made difficult by the large standard errors (rg BW_251dph_ – BW_980dph_ = 0.13 ± 0.38, rg TL_251dph_ – TL_980dph_ = 0.04 ± 0.39).

#### 3.3.2. Heritability of Flesh Composition

**Figure 2:**
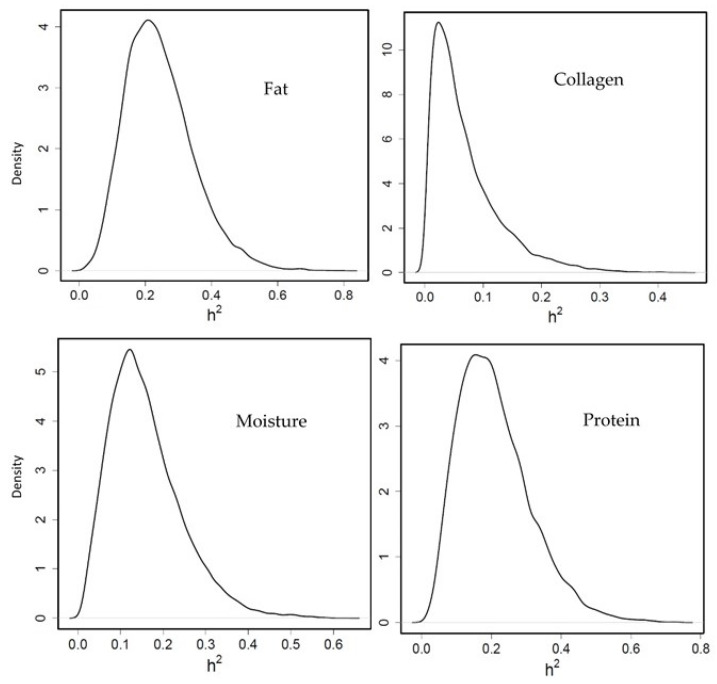
Posterior marginal distribution of heritabilities of fillet fat, collagen, moisture and protein percentage in gilthead seabream. h2 = heritability. (*n* = 399).

Moderate heritability was obtained for fillet fat percentage (0.24 [0.06–0.44] PM and HPD in brackets, Figure 2) which agrees with Elalfy et al. [27] and García-celdrán et al. [26], who showed 0.27 and 0.31, respectively. In our study, protein percentage heritability was moderate (0.21 [0.03–0.41]), however García-celdrán et al. [26] and Elalfy et al. [27] reported low protein heritability (0.03 and 0.08, respectively). Regarding the collagen percentage, the heritability was low (0.06 [0.002–0.19]) in this study, similar to that in García-celdrán et al. [26] (0.03) and Navarro et al. [43] (0.02). The moisture percentage showed a medium genetic heritability (0.15 [0.015–0.32]) in the present investigation, however it has been reported with considerable variation between other studies ranging from medium to low heritabilities, such as Garcia-celdrán et al. [26] and Elalfy et al. [27] (0.24 and 0.29, respectively) and Navarro et al. [43] (0.09).

It is interesting to know the genetic correlation between BW and fat percentage, since most breeding programs select fish to improve their growth. In our study, the genetic correlation between both traits was not estimated with precision because of the limited data available. When this correlation was estimated [26,27], a positive medium-high genetic correlation was observed, indicating that when fish are selected by growth, their fillet fat percentage increased indirectly.

#### 3.3.3. Heritability of Fatty Acid Profile

**Figure 3:**
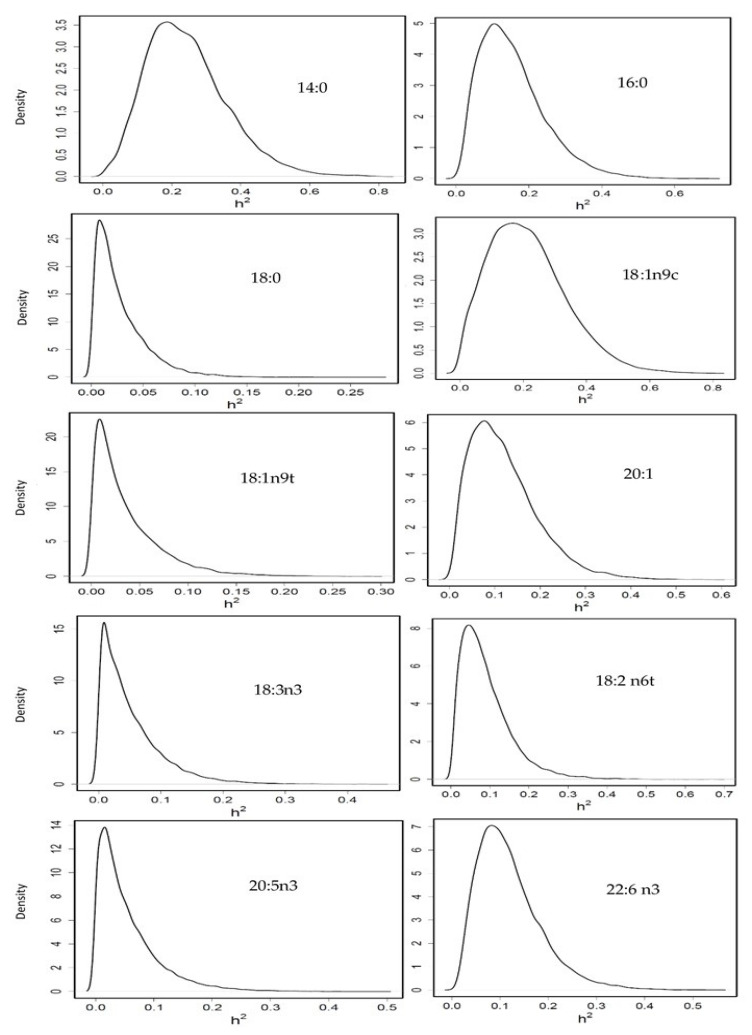
Posterior marginal distribution of heritabilities of fatty acids profile in gilthead seabream. h2 = heritability. *n* = 399.

Heritability was moderate for 14:0 (0.24 [0.04–0.48]), 16:0 (0.15 [0.01–0.33]) and 18:1n9c (0.20 [0.005–0.43]); it was low for 20:1 (0.12 [0.01–0.26]), and 22:6n3 (0.11 [0.017–0.25]); and practically null for 18:0 (0.02 [0.00–0.07]), 18:1n9t (0.03 [0.00–0.10]), 18:2n6t (0.09 [0.00–0.21]), 18:3n3 (0.05 [0.00–0.15]) and 20:5n3 (0.05 [0.00–0.15]) (Figure 3) and 20:4n6 (0.03 [0.00–0.10]), 16:1 (0.04 [0.00–0.12]) and 22:1 (0.06 [0.00–0.16]) although the density plots for these last three FA are not represented.

**Figure 4:**
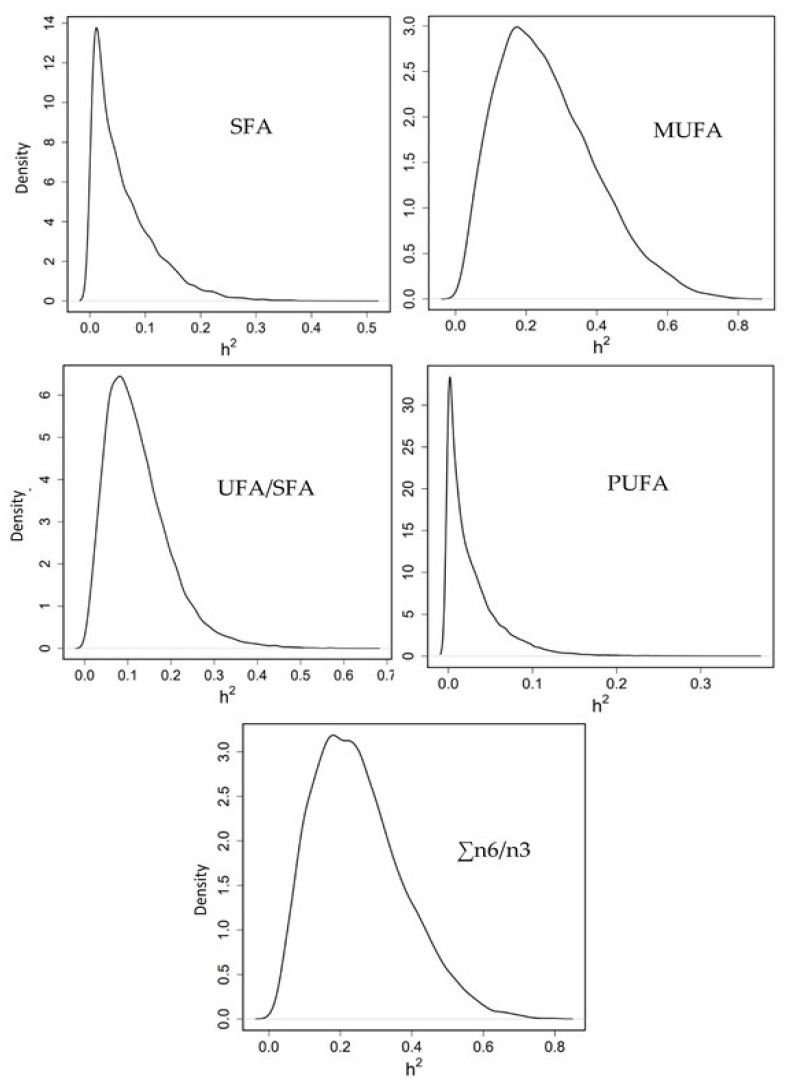
Posterior marginal distribution of heritabilities of SFA: saturated fatty acids, MUFA: monounsaturated fatty acids, PUFA: polyunsaturated fatty acids, UFA: unsaturated fatty acids and the ratio omega 6/omega 3 fatty acids in gilthead seabream. h2 = heritability. 399 data.

Heritability for the summatory of SFA (0.06 [0.00–0.17]) and PUFA (0.02 [0.00–0.09]) was almost zero; low for the ratio UFA/SFA (0.12 [0.01–0.26]); and medium for MUFA (0.26 [0.03–0.53]) and n6/n3 ratio (0.25 [0.03–0.49]) (Figure 4).

To our knowledge, there is no study about genetic parameters of FA in gilthead seabream. In Nile tilapia, the heritabilities for SFA were generally moderate, and for MUFA, PUFA and for total SFA, total MUFA, total PUFA, n3/n6 and UFA/SFA were low [29]. In Atlantic salmon, flesh n3 LCPUFA composition was highly heritable (h2 = 0.77 ± 0.14) and the authors [28] observed that families with a high percentage of n3 LCPUFA in flesh presented higher expression for genes related to hepatic lipid transport, and implicated increased activity of a transcription factor, hepatic nuclear factor 4α (HNF4α), possibly as a result of family differences in transforming growth factor b1 (Tgfb1) signalling. In that study, the authors [28] also highlighted that FLC was highly and negatively correlated with percentage n3 LCPUFA (−0.77), and FLC was positively correlated to BW. In Nile tilapia [29], the genetic associations of the PUFA group (20:5n3 and C18:3n6) with BW traits were strongly negative (−0.55 to −0.78); and for two SFA the genetic correlations of 18:0 and 24:0 with fillet fat percentage were negative (−0.11 and −0.85, respectively). In our study, genetic correlations between FA and fillet fat percentage could not be estimated with precision, likely due to limited data availability. However, phenotypic correlation PUFA-Fillet fat percentage was significantly negative (−0.12). A major part of LCPUFA is in the membrane phospholipids (PL), which presents an upper threshold, because amounts of PL molecules in tissue are likely determined by a volume of membranes. Thus, when the level of fattening increases in a fish, most of that fat is deposited in muscles, as TAG, to be an energy reserve. Thus, when FLC increases, fat deposits increase, TAG content increases and PL, together LCPUFA, is diluted [21].

In shrimp [44], limited heritabilities for FA were estimated; nevertheless, some important FA, such as DHA had significant variance among families with similar heritability (0.12 ± 0.06) to our study. In accordance with us, ARA, which is tightly linked to the immune response, showed a heritability not significantly different from zero.

Therefore, considering the positive genetic correlation between growth and fillet fat content, and the negative genetic correlation between fillet fat content and PUFA percentage, breeding for fish with higher growth is expected to cause an increase in the fillet fat percentage and a decrease of its PUFA percentage. In addition, most of the SFA and oleic, DHA, MUFA and the ratio n6/n3 have been shown to be heritable traits, thus their analyses should be considered in a breeding program.

The measurement of FA is expensive and time consuming, therefore further studies should be continued to investigate the relation between fillet fat content and its FA profile. The Fish Fat Meter device (Distell.com, West Lothian, Escocia) has been developed as a non-invasive tool to measure flesh fat content and a high correlation with FLC [27] has been demonstrated, thus it could be used as an easy non-invasive measurement.

## 4. Conclusions

Breeding programs in gilthead seabream usually include growth as the first criterion in the selection process. However, other quality traits, such as fillet fat percentage and its fatty acids profile should be considered, since they are very important traits for the consumer from a nutritional point of view. In addition, these quality traits are also related to the fish immune system and, consequently, to its disease resistance. Further studies to investigate the consequences of selecting fish for growth based on their fat content and their fatty acids profile are advisable.

## Figures and Tables

**Table 1:** Fatty acid (FA) composition of the diets (% total FAME), mean ± standard error.

FA	D4	D6
14:0	2.91 ± 0.09	1.89 ± 0.04
16:0	15.5 ± 0.11	11.4 ± 0.01
18:0	5.16 ± 0.34	4.31 ± 0.02
SFA	24.3 ± 0.09	17.5 ±0.08
16:1	4.05 ± 0.08	2.66 ± 0.00
18:1 *	29.9 ± 0.18	42.9 ± 0.30
C 20:1	3.03 ± 0.08	2.38 ± 0.08
C 22:1	2.05 ± 0.32	1.17 ± 0.15
MUFA	39.2 ± 0.13	49.7 ± 0.54
18:2n6	18.9 ± 0.21	20.1 ± 0.31
18:3n3	4.61 ± 0.26	6.05 ± 0.03
20:4n6	1.18 ± 0.59	0.00 ± 0.00
20:5n3	4.58 ± 0.03	2.81 ± 1.40
22:6n3	6.19 ± 0.52	4.01 ± 0.06
PUFA	36.4 ± 0.04	32.6 ± 0.45

SFA: saturated fatty acids, MUFA: monounsaturated fatty acids, PUFA: polyunsaturated fatty acids. 18:1 * refers only to n9c, because n9t was almost null.

**Table 2:** Phenotypic results (least square means ± standard error) for body weight, fork length, and flesh composition for gilthead seabream.

Offspring				Covariate BW_980dph_	
Traits	*n*	Mean	S.E.	b	S.E.
BW_251dph_ (g)	392	43.7	0.95	NI	
TL_251dph_ (cm)	392	13.8	0.09	NI	
BW_980dph_ (g)	392	450.2	4.14	NI	
TL_980dph_ (cm)	392	28.7	0.11	NI	
Fat (%)	389	10.1	0.12	0.006 *	0.002
Collagen (%)	388	1.74	0.02	0.001 *	0.000
Moisture (%)	389	64.6	0.11	−0.007 *	0.001
Protein (%)	389	19.8	0.06	<0.000	0.001

BW_251dph_ and BW_980dph_ = body weight at 251 dph and 980 dph respectively; TL_251dph_ and TL_980dph_ = total length at 251 dph and 980 dph respectively; * = covariate was significant (*p* < 0.05). NI = not included.

**Table 3:** Main fatty acids (FA, expressed as %) of gilthead seabream flesh adjusted to 10.1 fat percentage.

FA	Offspring			Covariate Fat Percentage	
	*n*	LSM	S.E.	b	S.E.
14:0	394	3.29	0.03	0.011	0.015
16:0	371	17.9	0.23	0.018	0.090
18:0	392	4.86	0.11	−0.019	0.045
SFA	397	28.3	0.30	−0.002	0.130
16:1	397	0.32	0.04	−0.018	0.015
18:1n9c	392	33.6	0.70	0.232	0.277
18:1n9t	333	3.55	0.11	−0.005	0.047
20:1	392	2.38	0.03	−0.004	0.014
22:1	397	0.80	0.02	−0.014	0.008
MUFA	397	44.38	0.57	0.291	0.209
18:2n6	334	15.6	0.21	−0.054	0.084
18:3n3	389	3.77	0.05	0.055 *	0.022
20:4n6	399	0.63	0.02	−0.017	0.006
20:5n3	381	2.88	0.04	0.008	0.016
22:6n3	384	6.84	0.12	−0.097 *	0.048
PUFA	397	29.84	045	−0.400	0.165
∑ n6/n3	394	1.05	0.35	0.249	0.140
UFA/SFA	392	2.97	0.08	−0.029	0.034

LSM: least square mean, SE: standard error; SFA: saturated fatty acids, MUFA: monounsaturated fatty acids, PUFA: polyunsaturated fatty acids, UFA: unsaturated fatty acids; * = covariate was significant (*p* < 0.05).
